# The TASK-1 and TASK-3 activator JG-C3-98 attenuates cold and mechanical responses in primary somatosensory neurons

**DOI:** 10.3389/fphar.2026.1844406

**Published:** 2026-06-09

**Authors:** Pedro De-la-Torre, Miguel Valencia, Jaime Gálvez, Victoria Flores del Pino, Ana Gómez del Campo, Sofía Romero, Elizabeth Mendoza, Susanne Rinné, Aytug Kiper, Luis Prent, Ariela Vergara-Jaque, Yuliet Mazola, Christian Olivera-Fuentes, Rafael Zúñiga, José C. E. Márquez Montesinos, Benjamín Rojas, Luciano Peña, Jairo Quiroga, María Pertusa, Niels Decher, Leandro Zúñiga, Wendy González, Rodolfo Madrid

**Affiliations:** 1 Department of Otolaryngology - Head and Neck Surgery, Harvard Medical School and Mass Eye and Ear, Boston, MA, United States; 2 Universidad Simón Bolívar, Barranquilla, Colombia; 3 Facultad de Ciencias Básicas, Universidad del Atlántico, Barranquilla, Colombia; 4 Universidad de la Costa - CUC, Barranquilla, Colombia; 5 Caribe Therapeutics, Barranquilla, Colombia; 6 Departamento de Biología, Facultad de Química y Biología, Universidad de Santiago de Chile, Santiago, Chile; 7 Faculty of Engineering, School of Food Engineering, Universidad del Valle, Tuluá, Colombia; 8 Institut für Physiologie und Pathophysiologie, Philipps-Universität Marburg, Marburg, Germany; 9 Institute of Physiology, University Medicine Greifswald, Greifswald, Germany; 10 Departamento de Ciencias Químicas, Facultad de Ciencias Exactas, Universidad Andrés Bello, Viña del Mar, Chile; 11 Center for Bioinformatics, Simulations and Modelling, Universidad de Talca, Talca, Chile; 12 Laboratorio de Fisiología Molecular, Facultad de Medicina, Universidad de Talca, Talca, Chile; 13 Center for Nanomedicine, Diagnostic, and Drug Development (ND3), Facultad de Medicina, Universidad de Talca, Talca, Chile; 14 Heterocyclic Compounds Research Group, Department of Chemistry, Universidad del Valle, Cali, Colombia; 15 Center for the Development of Nanoscience and Nanotechnology (CEDENNA), Proyecto CIA250002, Facultad de Medicina, Universidad de Talca, Talca, Chile

**Keywords:** K2P channels, nociceptive behavior, pain, peripheral neuropathies, pharmacology of K2P channels

## Abstract

K2P channels underlie background K^+^ currents that act as brakes on neuronal excitability. In the somatosensory system, the K2P channels TASK-1 and TASK-3 play a relevant role in cold sensing, mechanosensitivity, and pain. Combining molecular docking and molecular dynamic simulations, patch-clamp recordings of recombinant channels, Ca^2+^-imaging and patch-clamp analysis in cultured primary somatosensory neurons from trigeminal and dorsal root ganglia, extracellular recording of the nerve endings of trigeminal neurons at the corneal surface, and behavioral analysis in mice models of acute, irritative, and neuropathic pain, we described and characterized the novel and rationally designed activator of TASK-1 and TASK-3 channels JG-C3-98. In HEK293 cells, JG-C3-98 showed a strong activation effect on TASK-1 and TASK-3, but not on TRAAK, TREK-1, TREK-2, and TRESK. In cultured primary somatosensory neurons, we have found that JG-C3-98 shifted the thermal threshold of cold thermoreceptor neurons to lower temperatures and reduced the maximal mechanically evoked responses in neurons responding to hypoosmotic stimulation. In *ex vivo* recordings of corneal cold-sensitive neurons, JG-C3-98 also shifted the thermal threshold of these nerve endings to lower temperatures. In a group of cultured dorsal root ganglia neurons, electrophysiological analysis suggests that JG-C3-98 reduces excitability and activates an outward current compatible with TASK-1/3 channels. Besides, intraplantar administration of a single dose of JG-C3-98 in the hind paw increases the threshold of the mechanically evoked pain sensitivity and reduces AITC-evoked nociceptive responses in mice. Importantly, in a model of neuropathic pain induced by chronic constriction of the sciatic nerve, we have found that cold allodynia resulting from this form of peripheral nerve damage is also reduced by intraplantar administration of a single dose of JG-C3-98 in the hind paw. Altogether, these results suggest that JG-C3-98 could be a new and potentially effective pharmacological tool for reducing cold and mechanical sensitivity across different somatosensory territories, both in physiological and pathophysiological conditions, and serve as a molecular scaffold for developing novel, more effective antinociceptive compounds that specifically act as TASK channels activators.

## 1 Introduction

The two-pore domain potassium (K2P) channels underline background K^+^ currents that stabilize the resting membrane potential of excitable and non-excitable cells (Gada and Plant, 2019; Mathie et al., 2021). As such, their activation in neurons contributes to the fine-tuning of membrane excitability, acting as molecular brakes on electrical responses. These channels are grouped into six subfamilies (TWIK, TREK, TASK, TALK, THIK, and TRESK), encompassing the 15 mammalian K2Ps. The general structure of K2P channels includes four transmembrane domains (TM1-TM4), two pore-forming domains (P1 and P2), and a cap structure formed by the extracellular TM1-P1 linkers. Functional K2P channels are mainly homodimers, but some members have been shown to form heterodimers between subfamilies (Lengyel et al., 2020; Khoubza et al., 2021; Khoubza et al., 2022; Rinné et al., 2024). Although these channels are generally considered voltage-independent, some K2Ps exhibit voltage-dependent conductances (Schewe et al., 2016).

In primary sensory neurons from trigeminal ganglia (TG) and dorsal root ganglia (DRG), thermal, chemical, and mechanical transduction, and the subsequent increase in the action potential firing, result from the concerted action of several classes of ion channels. These background, transduction, and voltage-gated channels functionally coexist to give shape to the net electrical response to peripheral stimulation (Vriens et al., 2014). Among them, several members of the K2P channels family play a critical role in the excitability of primary afferents and in pain neurophysiology, under both physiological and pathophysiological conditions (Bayliss and Barrett, 2008; Plant, 2012; Gada and Plant, 2019; Smith, 2020). Due to their relevance, these channels have been considered promising pharmaceutical targets for developing more effective pain-drug modulators with the potential to tackle pain at its source.

A key group of K2Ps in the physiology of primary sensory neurons is the TASK channels. TASK-1 and TASK-3 channels are expressed in nociceptive neurons, and recent evidence has demonstrated their emerging role in pain physiology (Fan et al., 2022). TASK-1 knockout mice display thermal hyperalgesia in the hot-plate test, emphasizing the importance of this channel in regulating nociception (Linden et al., 2006). Besides, it has been shown that the expression of TASK-3 and TWIK-1 channels is downregulated in response to a neuropathic lesion, with no significant changes in TASK-1 expression (García et al., 2019). TASK-3 channels are highly co-expressed with the cold-activated TRPM8 channel in primary somatosensory neurons, which accounts for the observation that cold-sensitive neurons from TASK-3 knockout mice exhibit a ∼2 °C shift in thermal threshold toward warmer temperatures, resulting in cold allodynia in these animals (Morenilla-Palao et al., 2014). Notably, this shift mirrors that observed in neurons from allodynic mice following peripheral nerve injury in different somatosensory territories (González et al., 2017; Piña et al., 2019; Piña et al., 2024). Thus, to prevent the change in thermal sensitivity toward warmer temperatures that leads to cold-evoked allodynia, activating these TASK channels above their basal levels could revert this altered phenotype by counteracting the depolarizing effect of TRPM8 in response to cold stimulation. Furthermore, enhancing TASK channel activity may serve as a general strategy to mitigate the exacerbated excitability of primary afferents in several damage-triggered painful sensory alterations.

Some K2P channels can be activated by volatile anesthetics such as halothane and isoflurane, which show low K2P-channel selectivity. At high concentrations, isoflurane activates TASK-3 and the heterodimeric TASK-1/TASK-3 channels, but inhibits TASK-1 (Berg et al., 2004). The antifungal drug terbinafine has also been reported to activate the TASK-3 channel, although it exhibits significant side effects (Wright et al., 2017; Ren et al., 2020). Interestingly, the peripherally acting compound CHET3 is a selective TASK-3 agonist that attenuates thermal hyperalgesia and mechanical allodynia in rodent pain models, with no effect on TASK-1 channels (Liao et al., 2019). In this scenario, targeting TASK-1 and TASK-3 channels simultaneously with a single molecule represents a potential therapeutic strategy to modulate the altered excitability of primary sensory afferents, thereby reducing painful thermal and mechanical hypersensitivity in pathological states.

In this study, we discovered and characterized the JG-C3-98 compound as a selective and effective activator of the TASK-1 and TASK-3 channels. Using computational, *in vitro*, *ex vivo*, and *in vivo* approaches, we have explored the effect of JG-C3-98 on cold and mechanical sensitivity in primary somatosensory neurons and tested the efficacy of this new TASK-1/TASK-3 activator in nociceptive, irritative, and neuropathic pain models.

## 2 Materials and methods

### 2.1 Synthesis and characterization data for ethyl (2R,3R,4S,5R)-1-benzyl-5-(5-methylthiophen-2-yl)-4-nitro-3-phenylpyrrolidine-2-carboxylate (JG-C3-98)

5-methylthiophene-2-carboxaldehyde (100.0 mg, 1.0 eq.), *trans*-β-nitrostyrene (1.0 eq.), *N*-benzylglycine ethyl ester (1.5 eq.) and toluene (3 mL) were added into a round bottom flask equipped with a magnetic stirring bar. The mixture was heated under reflux for about 12 h. The reaction was monitored by thin-layer chromatography TLC. After the reaction was completed, the mixture was cooled to room temperature, and the solvent was removed under reduced pressure. The resulting crude product was purified using column chromatography with silica gel using a mixture of dichloromethane/hexane (7:3) as eluent. A racemic mixture of stereoisomers was obtained and characterized by nuclear magnetic resonance as well as spectroscopic and spectrometric analysis.

### 2.2 Molecular docking

Ligand JG-C3-98 was processed using the LigPrep module (Schrödinger Release 2020-3: LigPrep, Schrödinger, LLC, New York, NY, 2020), incorporating charges and parameters based on the OPLS2005 force field (Kaminski et al., 2001; Banks et al., 2005; Shivakumar et al., 2010) and determining protonation states at pH 7.0 using Epik (Shelley et al., 2007; Greenwood et al., 2010). The protein structures of hTASK-1 (PDB: 6RV2, (Rödström et al., 2020)) and hTASK-3 (PDB: 8K1V, (Lin et al., 2024)) were refined by completing missing residues, accurately assigning side-chain protonation states at pH 7.0 using PropKa (Olsson et al., 2011; Søndergaard et al., 2011), and optimizing potential energy with the OPLS2005 force field (Kaminski et al., 2001; Banks et al., 2005; Shivakumar et al., 2010) via the Protein Preparation Wizard (Madhavi Sastry et al., 2013). Molecular docking was performed in hTASK-1 and hTASK-3, with the docking grid (30 × 30 × 30 Å^3^) centered on the mass of the Halothane response element (Talley and Bayliss, 2002) using Schrödinger’s Glide module (Friesner et al., 2004; Halgren et al., 2004; Farid et al., 2006) in extra precision (XP) mode (Friesner et al., 2006). Poses were visually inspected, and the final pose was selected based on the lowest XP score and proximity to Halothane response element residues.

### 2.3 Molecular dynamic simulation

Molecular dynamics (MD) simulations were carried out using the Maestro suite (Schrödinger Release 2020-3: Maestro, Schrödinger, LLC, New York, NY, 2020), utilizing the complexes obtained from the molecular docking process and incorporating structural waters and Cholesteryl succinate from each PDB file. Channel-ligand complexes were embedded in a pre-equilibrated 1-palmitoyl-2-oleoyl-sn-glycero-3-phosphocholine (POPC) bilayer membrane model, aligned beforehand using the OPM server (Lomize et al., 2012). The systems were then solvated with the single point charge (SPC) water model. To neutralize each system and achieve a 0.15 M ion concentration, K^+^ and Cl^−^ ions were included.

Ion distribution in the selectivity filter (SF) followed a soft knock-on approach, placing K^+^ ions at S2 and S4, while water molecules occupied S1 and S3. MD simulations were performed in an NPγT ensemble with a surface tension of 0.0 bar Å using OPLS2005 force field (Kaminski et al., 2001; Banks et al., 2005; Shivakumar et al., 2010). The Nosé-Hoover Chain thermostat (Cheng and Merz, 1996) and the Martyna-Tobias-Klein barostat (Martyna et al., 1994) maintained stable temperature and pressure conditions at 300 K and 1.01325 bar, respectively, with an integration time step of 2 fs. The initial equilibration phase lasted 20 ns, during which positional restraints of 1.0 kcal × mol^-1^ × Å^-2^ were applied to the secondary structure of the protein, ligand atoms, ions, and water molecules. In the subsequent production phase of the MD simulation, all previously imposed positional restraints were removed. Each system underwent three independent MD simulations, each running for 100 ns, resulting in a cumulative 300 ns of MD production for each holo system channel.

### 2.4 Non-bonding interactions of JG-C3-98

To characterize the binding mode of the compound JG-C3-98 in the TASK-1 and TASK-3 channels, two computational tools were employed: Protein-Ligand Interaction Profiler (PLIP) (Salentin et al., 2015; Adasme et al., 2021) and GetContacts (https://getcontacts.github.io/). For each molecular dynamics simulation, 1000 frames were extracted, considering the ligand, residues, and water molecules located within ≤5 Å of the ligand. Default parameters were used in both computational tools for the automatic detection of interactions. Finally, for the generation of heatmaps, equal weight was assigned to each type of interaction per residue. Contact frequencies were normalized with respect to the maximum value observed in each protein-ligand system.

### 2.5 Electrophysiological recordings in HEK-293 cells

The human embryonic kidney cell line HEK-293 was acquired from the American Type Culture Collection (Manassas, VA, United States of America). HEK-293 cells were cultured in Dulbecco’s modified Eagle’s Medium nutrient mixture (DMEM/F-12) (Invitrogen Life Technologies, Carlsbad, CA, United States of America) supplemented with 10% (v/v) fetal bovine serum (FBS) (Thermo Fisher Scientific, Waltham, MA, United States of America) and 1% antibiotics (100 U/mL penicillin and 100 μg/mL streptomycin) (Cytiva HyClone, Thermo Fisher Scientific). Cells were maintained in a humidified incubator at 37 °C and 5% CO_2_ atmosphere. For the electrophysiological experiments using K2P channels, HEK-293 cells were transfected with cDNAs encoding hTRAAK (KCNK4) obtained from Addgene (plasmid #133080), hTREK-1 (KCNK2, EF165334), hTREK-2 (KCNK10, EU978938), hTASK-1 (KCNK3, NM_002246), hTASK-3 (KCNK9, AF212829), hTRESK (KCNK18, NM_181840), and the hTASK-3/L247M mutant.

Co-transfections of plasmids containing cDNAs of interest and a reporter vector encoding the cDNA for green fluorescent protein (GFP) (1–2 μg of DNA plasmid) were achieved with a 3:1 ratio (K2P channel plasmid: GFP plasmid) using Xfect polymer (Clontech, Mountain View, CA, United States of America). The cells were incubated for 4 h in transfection medium OptiMEM (Invitrogen Life Technologies, Carlsbad, CA, United States of America). After incubation, the medium was exchanged with fresh culture medium and maintained at 37 °C with 5% CO_2_ for 12 h before electrophysiological measurements were made. All reported studies were performed in at least three independent experiments, with replicate transfections in each experiment. hTREK-2, hTASK-1, and hTASK-3 constructs were a kind gift from Dr. Steve Goldstein (University of California, Irvine, CA, United States of America). The cDNA encoding hTREK-2 was generously provided by Dr. Dierk Thomas (University of Heidelberg, Germany). The hTRESK construct was a generous gift from Dr. Péter Enyedi (Semmelweis University, Budapest, Hungary). hTASK-3/L247M mutant in pSGEM vector was built in our group at Marburg University, Germany. For the expression in mammalian cells, hTASK-3/L247M mutant was subcloned into pCR3.1 vector.

Whole-cell currents were recorded from HEK-293 cells transiently transfected with different K2P-encoding plasmids of wild-type or mutant channels at room temperature 24–48 h post-transfection using a PC-501A patch clamp amplifier (Warner Instruments, Hamden, CT, United States of America), as described previously (Zúñiga et al., 2011). The pClamp10 software (Molecular Devices, San Jose, CA, United States of America) with an acquisition card (DigiData 1440, Molecular Devices, San Jose, CA, United States of America) was used for voltage protocols and data acquisition. Glass microelectrodes (3–5 MΩ) were made from borosilicate pipettes using P97 Flaming/Brown Micropipette Puller (Sutter Instruments, Novato, CA, United States of America). The cells were continuously perfused with bath solution containing (in mM): 135 NaCl, 5 KCl, 1 MgCl_2_, 1 CaCl_2_, 10 HEPES, and 10 Sucrose, adjusted to pH 7.4 or 8.0 with NaOH. Intracellular pipette solution contained (in mM): 145 KCl, 2 MgCl2, 5 EGTA, and 10 HEPES, adjusted to pH 7.4 with KOH.

The JG-C3-98 compound was dissolved in dimethyl sulfoxide (DMSO) to obtain 200 mM stock solutions. A working concentration of 100 μM JG-C3-98 compound was freshly prepared by diluting the stock solution with the bath solutions to obtain the desired concentration. For TASK-1 only, the JG-C3-98 compound was specifically dissolved in the bath solution at pH 8.0 to minimize proton-mediated inhibition and ensure the accurate and reproducible measurement of maximal outward currents. Cells were held at −80 mV, and then currents were recorded using a protocol of 500 ms of duration from −100 to +100 mV with increments of 10 mV, or starting from a holding potential of −80 mV and using 20 mV depolarizing voltage steps of 100 ms followed by a final 100 ms ramp to −120 mV. After stable current amplitudes were reached, cells were exposed to JG-C3-98 compound, which was applied via the bath solution. Currents were recorded continuously during repeated stimulation until the compound induced a steady-state effect. For all channels, the effect of JG-C3-98 compound was analyzed at +80 mV, except for TREK-2, which was analyzed at +40 mV because of the large outward current amplitudes.

Current amplitudes before (*I*_control) and after (*I*_compound) the application of the compound was compared to evaluate its effect. The percentage of effect was calculated using the formula: Effect (%) = 100 × (*I*_ compound − *I*_control)/*I*_control. Voltage protocols, patch-clamp acquisition, and analysis were conducted using WinWCP software (University of Strathclyde). Data analysis was performed using SigmaPlot version 12.0 (Systat Software Inc., San Jose, CA, United States of America). To determine EC_50_ values, concentrations of 0.01 μM, 0.1 μM, 1 μM, 10 μM, 50 μM, and 100 μM JG-C3-98 compound were prepared for TASK-1 and TASK-3 channels. The EC_50_ experiments were repeated at least three times with five replicates in each experiment. Curves were fitted to a four-parameter logistic function and were constructed by using the average of the fitted parameters of the individual experiments.

For the recordings of TRPM8 channels, HEK-293 cells transfected with mTRPM8 were used. Twenty-four hours before patch-clamp experiments, cells were trypsinized and seeded on poly-l-lysine-coated 6 mm #0 glass coverslips (Menzel-Gläser, Braunschweig, Germany). Whole-cell patch-clamp recordings in transfected HEK-293 cells were performed simultaneously with temperature recordings. Standard patch pipettes (3–5 MΩ) were made of GC150F-7.5 borosilicate glass capillaries (Harvard Apparatus) and contained the following (in mM): 130 CsCl, 1 EGTA, 10 HEPES, 4 ATP-Mg, and 0.4 GTP-Na, pH adjusted to 7.4 with CsOH. The bath solution was the same as in the Ca^2+^ imaging experiments and patch-clamp recordings in primary sensory neurons (see below). Current signals were recorded with an Axopatch 200B patch-clamp amplifier and digitalized using an Axon Digidata 1440A AD converter (Molecular Devices, San Jose, CA, United States of America). Stimulus delivery and data acquisition were performed using pClamp10 software (Molecular Devices, San Jose, CA, United States of America). Current-voltage (I–V) relationships were obtained from repetitive (0.2 Hz) voltage ramps (−100 to +180 mV, with a slope of 200 mV/s) as in (Rivera et al., 2020; Rivera et al., 2021).

### 2.6 Animals

This study was performed using young adults (P21-P90) male and female C57BL6 mice. The number of animals used in each assay was minimized in accordance with expected effect sizes from previous studies and/or power analysis to ensure adequate statistical significance while avoiding unnecessary animal use. Given the sample sizes and the representation of male and female animals in the experimental approaches, no sex-based analysis was performed, and data were pooled. Animals were housed in a 12 hour-light/dark cycle, with food and water *ad libitum*, and euthanized with CO_2_. All experiments were conducted according to the bioethical guidelines of the *Agencia Nacional de Investigación y Desarrollo (ANID)* and the Bioethical Committee of the University of Santiago de Chile (Protocol Reference Number 217.2024, 255.2024, and 029.2026).

### 2.7 Cell culture

Cultures of trigeminal and dorsal root ganglia neurons were prepared as (González et al., 2017; Piña et al., 2024). In brief, animals were euthanized by CO_2_ inhalation. After decapitation, sensory ganglia were removed and incubated in an enzymatic mixture in INC-mix solution (in mM: 155 NaCl, 1.5 K_2_HPO_4_, 10 HEPES, 5 Glucose, pH: 7.4) containing collagenase type XI (650 UI/mL; C7657, Sigma-Aldrich, St. Louis, United States of America) and dispase (5 UI/mL; 17105-041 GIBCO-Thermo Fisher Scientific, Waltham, MA, United States of America), during 40 min at 37 °C in 5% CO_2_. The sensory ganglia were then mechanically dissociated with polished Pasteur pipettes and the neurons were plated on poly-L-lysine-coated 6 mm #0 glass coverslips (Menzel-Gläser, Braunschweig, Germany), maintained in MEM media (Earle’s salts, 11095080, GIBCO-Thermo Fisher Scientific, Waltham, MA, United States of America) supplemented with MEM-vit (11120052, GIBCO-Thermo Fisher Scientific, Waltham, MA, United States of America), 10% FBS (SH30910.03, Hyclone, General Electric Healthcare Life Science, UT, United States of America), 200 μg/mL streptomycin, 125 μg/mL penicillin (15140-122, GIBCO-Thermo Fisher Scientific, Waltham, MA, United States of America), and used within 6–12 h for [Ca^2+^]_i_ imaging and patch-clamp recordings.

### 2.8 Ca^2+^ imaging

For ratiometric Ca^2+^ imaging experiments, sensory neurons were incubated in 5 µM Fura-2 AM (F1221, Invitrogen-Thermo Fisher Scientific, Waltham, MA, United States of America) in standard extracellular solution (containing in mM: 140 NaCl, 3 KCl, 1.3 MgCl_2_, 2.4 CaCl_2_, 10 HEPES, 10 glucose; 298 mOsm/kg, pH 7.4 adjusted with NaOH), supplemented with 0.02% Pluronic acid (P6867, Invitrogen-Thermo Fisher Scientific, Waltham, MA, United States of America) for 50 min at 37 °C, in darkness. Fluorescence measurements were obtained using an inverted Nikon Ti microscope equipped with a Super Plan Fluor ELWD ×20C objective N.A. 0,45 (Nikon Instruments Inc., Melville, NY, United States of America) and a 12-bit cooled ORCA C8484-03G02 CCD camera (Hamamatsu, Hamamatsu City, Japan). Fura-2 was excited at 340 and 380 nm with a Polychrome V monochromator (Till Photonics, Munich, Germany), with exposure times of 40 ms; the emitted fluorescence was filtered with a 510 nm long-pass filter. Calibrated ratios (0.5 Hz) were displayed online with HCImage v2 software (Hamamatsu, Hamamatsu City, Japan). Bath temperature (see details below) was sampled simultaneously using a BAT-12 microprobe thermometer (Physitemp Instruments, Clifton, NJ, United States of America) supplemented with an IT-18 T-thermocouple, using Clampex 10 software (Molecular Devices, San Jose, CA, United States of America). The signal was digitized with an Axon Digidata 1440A AD converter (Molecular Devices, San Jose, CA, United States of America).

Threshold temperature values for the rise in intracellular Ca^2+^ concentration ([Ca^2+^]_i_) were estimated as in (Rivera et al., 2021). For this, temperature was linearly interpolated at the midpoint between the baseline and the first point at which [Ca^2+^]_i_ elevation deviates by at least four times the standard deviation of the baseline. The increase in [Ca^2+^]_i_ in cultured cold sensitive neurons is due to Ca^2+^ entry through voltage-gated Ca^2+^ channels, which are activated during action potential firing. A very tight correlation between threshold temperature detected in the [Ca^2+^]_i_ signal and the threshold of action potential firing recorded in cell-attached mode (Viana et al., 2002; Madrid et al., 2009; González et al., 2015), allows the use of this non-invasive method to determine the thermal threshold of several cold-sensitive neurons simultaneously. For mechanical stimulation, we used brief pulses of a hypoosmotic extracellular solution (210 mOsm) to induce the activation of mechanosensitive channels by membrane stretching that produces transient and reversible increases in the [Ca^2+^]_i_ in cultured primary somatosensory neurons (Gomis et al., 2008).

### 2.9 Cell stimulation

Coverslips with plated sensory neurons were placed in a microchamber and continuously perfused with solutions at a rate of ∼1 mL/min at ∼34 °C, adjusted with a water-cooled computer-controlled CS-1 Temperature Controller (Cool Solutions Research Devices, Carrigaline, Ireland), with the outlet located on the imaging field and controlled by a feedback device. Cold sensitivity was investigated using a ∼30 s (s) ramp-like temperature drops to 20 °C from a basal temperature of 34 °C, either in control solution or in the presence of JG-C3-98 applied using the same perfusion system. Mechanical sensitivity was explored using 30 s pulses of hypoosmotic solution (210 mOsm/kg) with or without JG-C3-98. AITC-sensitivity in these neurons was explored using a 30 s pulse of 100 µM of this TRPA1 activator at the end of the experiment; positive responses were considered as an increase in the [Ca^2+^]_i_ of the neuron at 34 °C.

### 2.10 Patch-clamp in cultured primary sensory neurons

In current-clamp mode, DRG neurons were held at −60 mV and a series of negative and positive current steps of 500 ms duration (ΔI_ext_ = 10–100 pA, depending on input resistance) were delivered at a rate of 0.1 Hz to determine resting membrane potential, input resistance, rheobase current, spike duration and amplitude, inward rectification index, and I_ext_-evoked firing as in (González et al., 2017; Piña et al., 2024), in control conditions and in the presence of 100 µM JG-C3-98. Current-clamp recordings were performed simultaneously with temperature recordings. The same extracellular solution used in Ca^2+^ imaging experiments was used in patch-clamp recordings. Standard patch-clamp pipettes (4–5 MΩ resistance) were made with GC150F-7.5 glass capillaries (Harvard Apparatus, Holliston, MA, United States of America) and filled with intracellular solution containing (in mM): 105 K-gluconate, 35 KCl, 8.8 NaCl, 10 HEPES, 0.5 EGTA, 4 MgATP, 0.4 NaGTP, (300 mOsm/kg and pH 7.4, adjusted with KOH). Voltage and current signals were recorded using an Axopatch 200B and digitized with an Axon Digidata 1440A AD converter (Molecular Devices, San Jose, CA, United States of America). Stimulus delivery and data acquisition were performed using pClamp 10 software (Molecular Devices, San Jose, CA, United States of America). Before electrophysiological recordings, the thermal threshold in response to cold stimuli was determined using Ca^2+^ imaging.

### 2.11 Extracellular recordings of corneal cold thermoreceptor neurons

Extracellular recording of nerve terminal impulses (NTI) activity *in vitro* in mice was performed as in (Piña et al., 2019; Cornejo et al., 2020). In brief, eyes were carefully removed from the CO_2_ euthanized animals and placed in a 25 mL precipitated glass containing extracellular solution under constant oxygenation. Excised eyes were placed in the recording chamber, and the optic nerve and associated tissues were drawn into a suction tube at the bottom of the chamber. Eyes were continuously perfused (1 mL/min) with physiological saline solution containing (in mM): 128 NaCl, 5 KCl, 1 NaH_2_PO_4_, 26 NaHCO_3_, 2.4 CaCl_2_, 1.3 MgCl_2_, and 10 glucose, pH 7.4, continuously gassed with carbogen (95% O_2_, 5% CO_2_). The basal temperature of the bath solution was kept at ∼34 °C and was regulated using a CS-1 Temperature Controller (Cool Solutions Research Devices, Carrigaline, Ireland), controlled by computer, and the outlet was located close to the eye surface.

A glass pipette (tip diameter ∼50–100 µm) for recording extracellular NTI activity, filled with physiological saline solution, was positioned onto the corneal epithelium surface and slight suction was applied as we described in (Parra et al., 2010). Signals were amplified with an 1800 AC amplifier (A-M Systems, Carlsborg, WA, United States of America), and the data was acquired and analyzed using an Axon 1332A Digidata AD converter (Molecular Devices, San Jose, CA, United States of America) coupled to a computer running pClamp 9 software (Molecular Devices, San Jose, CA, United States of America). Further analysis was performed using Spike2 8.0 software (Cambridge Electronic Design, Milton, Cambridge, United Kingdom). Only nerve impulses that were readily distinguished from noise (∼10 µV peak-to-peak when low-pass filtered at 5 kHz) and with similar shape and amplitude were studied. Cold thermoreceptor nerve endings were identified by their typical spontaneous, often regular, low-frequency impulse activity at 34 °C, which increased during temperature reductions and were transiently silenced by re-warming. Ongoing action potentials firing (NTI activity) at 34 °C was recorded for at least 3 min prior to cooling. Basal mean ongoing activity (in impulses per s) was calculated during the 30 s preceding the onset of the first ∼30 s ramp-like temperature drop to 20 °C, at a rate of ∼0.7 °C/s. Cooling threshold was defined as the temperature value in degrees Celsius (°C) at which NTI frequency increased to a value that was the mean NTI frequency, measured during the 10 s period that preceded the onset of the cooling pulse, plus three times its standard deviation. This protocol was repeated each 5 min in control conditions and during exposure to JG-C3-98 after the first cold stimulus.

### 2.12 Intradermal injections

All subcutaneous injections into the plantar surface of the hind paw were performed using a 30-gauge needle coupled to a Hamilton syringe. For the AITC-evoked response experiments, 10 µL of 5 mM JG-C3-98, along with 10 mM allyl isothiocyanate (AITC) or vehicle (only AITC dissolved in ethanol (Marcotti et al., 2023)) was injected. To determine the effect of JG-C3-98 on the mechanical threshold, a single dose of 10 µL of 5 mM JG-C3-98 or vehicle (ethanol) was injected. For the acetone evaporative cooling test, injections of 5 µL of JG-C3-98 5 mM or vehicle were used.

### 2.13 Model of cold allodynia induced by axonal damage

We used peripheral axonal injury by chronic constriction of the sciatic nerve as a model of nerve damage manifesting cold allodynia (Bennett and Xie, 1988), as we did in (González et al., 2017). In brief, under ketamine and xylazine anesthesia, the right sciatic nerve was exposed after an incision to the skin and the separation of the heads of the *biceps femoris* muscle. At 5 mm (proximal) of the peroneal-tibial bifurcation, two loose silk ligatures with 8–0 chromic gut separated by 3 mm were tied around the exposed nerve, until approximately an 80% of its original diameter, and the incision was closed. For sham-operated animals, the sciatic nerve was exposed but not ligated.

### 2.14 Behavioral assays

Experiments were conducted during the light cycle. Mice were habituated to the behavioral room at least 3 hours prior to testing. Additionally, animals were acclimated to the experimental chambers on a mesh platform (hole size: 4 × 4 mm) for at least 20 min before injections. Investigators were blinded to both the surgical condition and the injected treatments during behavioral testing and subsequent video analyses.

### 2.15 Mechanical threshold (Von Frey test)

After the initial habituation and subsequent injection, animals were placed in a transparent box and allowed to acclimate for an additional 20 min. To assess mechanical sensitivity, calibrated Von Frey filaments BIO-VF-M (Bioseb Lab Instruments, Vitrolles, France) were used. Mechanical thresholds were determined using a modified version of the Dixon up-down method (Chaplan et al., 1994). Starting with the finest filament (1.65 mm), the thickness was increased with each measurement until a positive response (withdrawal or licking of the hind paw) was obtained. If the animal exhibited a positive response, the next finer filament was used in the subsequent measurement. Conversely, if a negative response occurred, the next thicker filament was used. The “up-down” paradigm was applied until at least six consecutive measurements exhibited an alternating pattern of positive and negative responses. The 50% response threshold of the last six measurements was calculated using the formula: 50% g threshold= (10Xf + kd)/10,000; where Xf is the size value of the last Von Frey filament applied, k is the correction factor based on the pattern of responses (Dixon’s calibration table), and d is the mean distance between the size of filament stimuli.

### 2.16 AITC-evoked nociceptive responses

Following the required habituation, animals were intradermally injected and immediately returned to the transparent Plexiglass box. After an extra acclimation of 5 minutes, aversive responses (time spent licking and lifting the injected paw) were recorded and quantified over a 10-min period.

### 2.17 Acetone evaporative cooling assay

Cold sensitivity was assessed with the acetone evaporation assay as in (González et al., 2017). Animals were placed in round plastic chambers on a mesh platform. A drop (∼20 µL) of acetone was applied to the plantar surface of the hind paw, and the number of the following nocifensive events were monitored over the following 60 s and an arbitrary score was assigned to the behavior: 0 indicated no response, 0.5 licking response, 1 flinching and brushing of the hind paw, 2 strong flinching, and 3 strong flinching, licking and paw guarding. Nocifensive responses were observed during the first minute after acetone application and measurements were repeated 3 times with a 10-min interval to obtain a mean value. The events for the contralateral paw measured under the same conditions were subtracted from the ipsilateral one to give the final net acetone score. A value of zero indicates no difference between cold-evoked sensations of the ipsilateral and contralateral hind paws whilst a positive value indicates cold allodynia.

### 2.18 Statistical analysis

Data are expressed as the mean ± SEM (standard error of the mean), except where indicated. Unless mentioned otherwise, when comparing two mean values, statistical significance was assessed using Student’s paired or unpaired two-tailed *t*-test, and the Mann-Whitney two-tailed test was used for nonparametric data. For multiple comparisons, one-way or two-way ANOVA was used with Dunnett or Bonferroni post hoc tests. If other tests were used, they were included in the main text and/or in the figure legends. Differences were considered significant when *p < 0.05, **p < 0.01, and ***p < 0.001. Besides where indicated, all exact p-values, statistical tests, and sample sizes are reported in the figure legends.

### 2.19 Reagents and drugs

Allyl isothiocyanate (AITC, 377430) was purchased from Sigma-Aldrich (St. Louis, MO, United States of America).

## 3 Results

### 3.1 JG-C3-98 is a strong and selective activator of TASK-1 and TASK-3 channels

**FIGURE 1:**
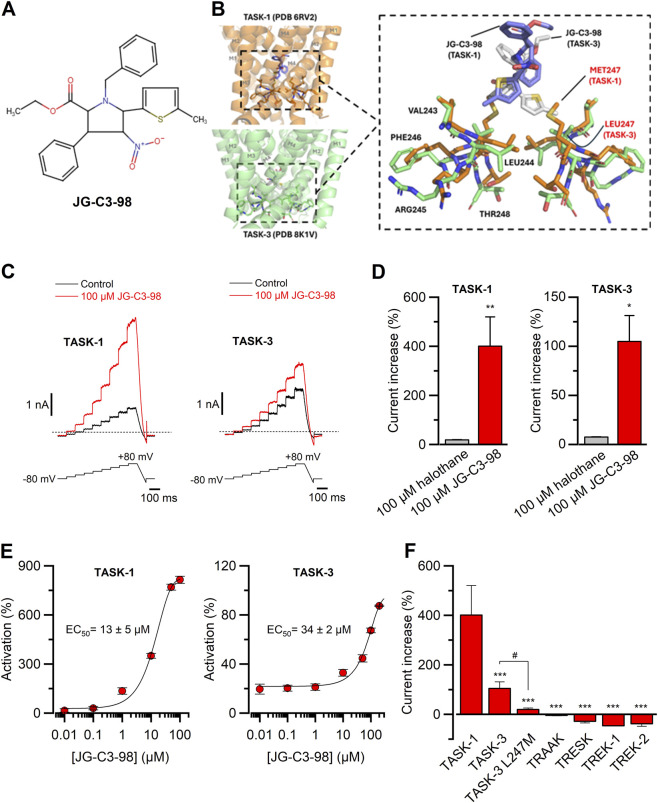
JG-C3-98 is a strong activator of TASK-1 and TASK-3 channels. **(A)** Structure of JG-C3-98 (Ethyl (2R,3R,4S,5R)-1-benzyl-5-(5-methylthiophen-2-yl)-4-nitro-3-phenylpyrrolidine-2-carboxylate). **(B)** *Left panel,* Structural representation of TASK-1 and TASK-3 channels, depicted as cartoon models in orange and green, respectively. The Halothane Response Element residues are shown in stick representation. The JG-C3-98 selected docking pose is visualized as a backbone stick, in purple (TASK-1) and white (TASK-3). The transmembrane domains of the TASK channels are indicated for structural context. *Right panel,* Close-up view of the alignment of the Halothane Response Element motif, including the docked JG-C3-98 from Panel A, represented in stick form. This alignment emphasizes the conservation of structural residues between TASK-1 (orange) and TASK-3 (green), with differential residues highlighted in red. Atoms are color-coded: nitrogen (blue), oxygen (red), and sulfur (yellow). **(C)** Representative whole cell recordings showing the effect of 100 μM JG-C3-98 on hTASK-1 (*left panel*) and hTASK-3 (*right panel*) channels expressed in HEK-293 cells. The stepwise increase currents were generated with the voltage protocol of initial hyperpolarization to −80 mV and then depolarization with 20 mV stepwise every 100 ms to +80 mV followed by a 100 ms ramp to −120 mV. **(D)** Bar graphs showing the effect of JG-C3-98 compared to halothane on the current of TASK-1 (left panel) and TASK-3 (right panel) channels expressed in HEK-293 cells, measured at +80 mV. Statistical analysis was carried out using Student’s t-test for unpaired samples (*p < 0.05; **p < 0.01). **(E)** Concentration-response curves of the activation of TASK-1 (left panel) and TASK-3 (right panel) expressed in HEK-293 cells. JG-C3-98 activation was analyzed at +80 mV. The EC_50_ values were 13 ± 5 μM and 34 ± 2 µM to hTASK-1 and hTASK-3, respectively. Curves are fit to a 4-parameter logistic function and were constructed by using the average of fitted parameters of the individual experiments; statistical analysis was made using the Kolmogorov-Smirnov test, Spearman’s rank correlation, and Durbin-Watson statistic. Data are shown as mean ± SEM (n ≥ 3). **(F)** Bar graph summarizing the effect of 100 µM JG-C3-98 on other key human K2P channels (hTASK-1, hTASK-3, the mutant hTASK-3L247M, hTRAAK, hTRESK, hTREK-1, and hTREK-2) in HEK-293 cells recorded at +80 mV. Data are shown as means ± SEM (n ≥ 3 for each channel). Statistical analysis was made using one-way ANOVA with Dunnett’s multiple comparison test (***p < 0.001); unpaired *t*-test with Welch’s correction was used to compare TASK-3 and hTASK-3 L247M mutant (^#^p = 0.0256).

In search of new TASK channel modulators, we adopted a rational design strategy inspired by conserved physicochemical features shared by previously described ligands, including aromatic moieties and nitrogen-containing heterocycles, which are thought to promote favorable hydrophobic contacts and polar interactions within TASK channel binding pockets (Bedoya et al., 2019). Based on this framework, we synthesized JG-C3-98, which proved to be a potent activator of TASK-1 and TASK-3 channels; a schematic representation of the synthesis process is shown in Supplementary Figure S1. The chemical structure of JG-C3-98 is shown in Figure 1A, while Figure 1B shows a structural representation of TASK-1 and TASK-3 channels with docked JG-C3-98. The study of non-covalent interactions between JG-C3-98 and the TASK-1 and TASK-3 channels using molecular dynamics simulations revealed significant contributions from residues L122 and L239 (Supplementary Figure S2), with these interactions being mainly hydrophobic. However, JG-C3-98 also establishes polar interactions with other residues, such as water bridges and hydrogen bonds. Overall, the main interactions with residues located within ≤5 Å of the ligand correspond to hydrophobic contacts (Supplementary Figure S3).

The activation of TASK-1 and TASK-3 channels by JG-C3-98 was verified by testing its effect on whole-cell currents in HEK-293 cells expressing these K2P channels. Representative whole cell recordings showing the effect of 100 μM JG-C3-98 on hTASK-1 and hTASK-3 channels are shown in Figure 1C. The currents were generated using a voltage protocol starting at −80 mV, followed by 20 mV depolarizing voltage steps of 100 ms, and a final 100 ms ramp to −120 mV (Figure 1C). Since the volatile anesthetic halothane is a known activator of both channels, the effect of 100 µM JG-C3-98 was compared with that of 100 µM halothane on channel activation at +80 mV in a separate group of cells. As shown in Figure 1D, JG-C3-98 induces a more substantial increase in the TASK-1 and TASK-3-dependent currents than this anesthetic agent at the same concentration. Besides, concentration-response curves for the activation of TASK-1 and TASK-3 channels by JG-C3-98 indicate that this compound exhibits greater potency and efficacy for TASK-1 than for TASK-3 (Figure 1E). To further explore its specificity at a saturating concentration, the effect of JG-C3-98 was also evaluated on other K2P channels involved in the electrical activity of primary afferents, including TRAAK, TRESK, TREK-1, and TREK-2 channels. We found that this compound showed strong subtype selectivity among these K2P channels expressed in HEK-293 cells, as TRAAK, TRESK, TREK-1, and TREK-2 were not activated by 100 µM JG-C3-98 (Figure 1F).

Finally, to investigate differences in affinity for JG-C3-98 between TASK-1 and TASK-3, we focused on the halothane response element (HRE), the binding site for volatile anesthetics that activate TASK channels, which we considered a putative docking site for JG-C3-98. From known activators (Supplementary Table S1), only halothane and TBE bind to both channels. The HRE sequences of TASK-1 and TASK-3 differ by a single residue (VLRF(M/L)T), with methionine present in TASK-1. Since L247 in TASK-3 (corresponding to M247 in TASK-1) is a relevant residue for drug binding, we explored the sensitivity of the TASK-3 channel containing the L247M mutation to JG-C3-98. We asked whether this substitution would increase the sensitivity of TASK-3 to JG-C3-98, as observed with other modulators that selectively affect TASK-1 over TASK-3. A1899, for example, is a ten-fold more potent blocker of TASK-1 than of TASK-3, and the TASK-1 M247L mutant shows a 3.3-fold increase in IC_50_ (Streit et al., 2011). In contrast, we found that JG-C3-98 was unable to activate the TASK-3-L247M mutant channel, suggesting that although this residue is essential for activation by this compound, it does not account for the differential affinity of JG-C3-98 between TASK-1 and TASK-3.

Altogether, these results suggest that JG-C3-98 is a novel activator of TASK-1 and TASK-3 channels, which may be used to explore the effect of activating these two key K2P channels in primary somatosensory neurons.

### 3.2 JG-C3-98 shifts the temperature threshold and reduces the mechanical responses of trigeminal neurons in culture

**FIGURE 2:**
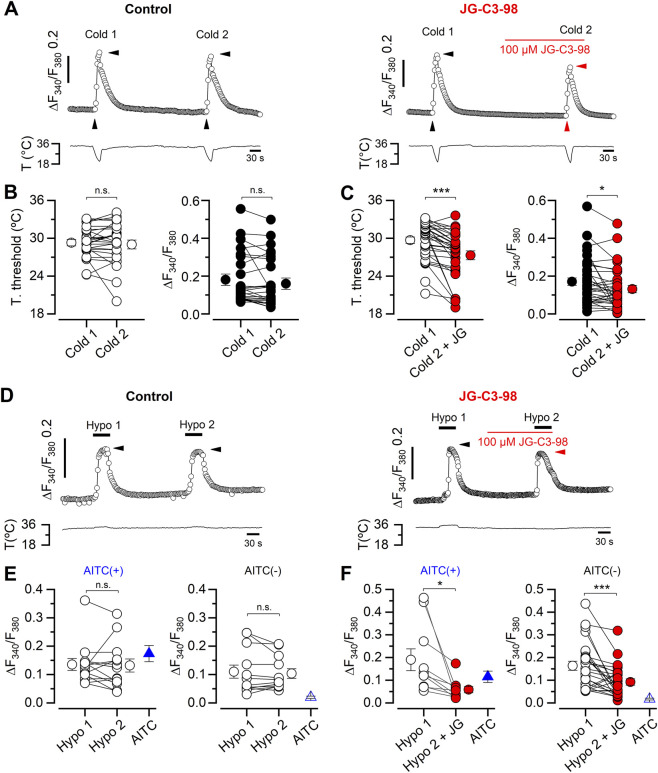
JG-C3-98 shifts the cold threshold to lower temperatures and reduces the mechanical responses of primary sensory neurons in culture. **(A)** Representative traces of [Ca^2+^]_i_ (as F_340_/F_380nm_ ratio) in cold-sensitive neurons with and without JG-C3-98 treatment. Two cold pulses were applied in control solution (left panel), while in the treatment condition, 100 µM JG-C3-98 was applied prior to and during the 2^nd^ pulse (right panel). Arrowheads indicate the thermal thresholds and maximal responses (control black, JG-C3-98 red). **(B,C)** Dot plots summarizing the individual and mean cold threshold (±SEM) and the individual and mean maximal response (±SEM) of cold-sensitive neurons in control condition (n = 27, n.s. p = 0.7027 and n.s. p = 0.0642, respectively), or treated with 100 µM JG-C3-98 (n = 33, ***p = 0.00003 and *p = 0.0133, respectively). **(D)** Representative traces of [Ca^2+^]_i_ (as F_340_/F_380nm_ ratio) in mechano-sensitive neurons with and without JG-C3-98. Two pulses of 210 mOsm/L (hypoosmotic) solution were applied in control condition (left panel) and in the presence of 100 µM JG-C3-98 before and during the second pulse (right panel). Arrowheads indicate the maximal responses (control black, JG-C3-98 red). **(E,F)** Dot plots showing the individual and mean maximal response (±SEM) to hypoosmotic solution in cultured neurons, sensitive and insensitive to 100 µM AITC (n = 13, n.s. p = 0.8262 in control, and n = 10, *p = 0.0212 in JG-C3-98, for AITC-sensitive neurons; and n = 12, n.s. p = 0.4522 in control, and n = 26, ***p = 0.0009 in JG-C3-98, for AITC-insensitive neurons). Filled and open blue triangles indicate the mean amplitude of the AITC-evoked response in AITC-sensitive and AITC-insensitive neurons, respectively. Statistical analysis was carried out using two tailed Student’s t-test for paired samples (n.s., p > 0.05; *p < 0.05; ***p < 0.001).

The temperature-sensitivity of cold thermoreceptor neurons depends on the TRPM8 channel (McKemy et al., 2002; Peier et al., 2002). Remarkably, TASK-3 channels are highly expressed in cold thermoreceptors, strongly modulating the thermosensitivity of these sensory fibers (Morenilla-Palao et al., 2014). We hypothesized that TASK-3 activation in cold-sensitive neurons could act as a molecular brake on their electrical activity, thereby reducing their thermosensitivity. With this idea in mind, we explored the influence of activating this K2P with JG-C3-98 in cold thermoreceptors in culture, examining the effect of this compound on their cold-evoked responses. Trigeminal cold-sensitive neurons in culture were identified by a rise in [Ca^2+^]_i_ during a cooling ramp using Ca^2+^ imaging (Madrid et al., 2006). We employed a double-pulse protocol, wherein we studied the response to cold before (Cold 1) and in the presence (or absence) of 100 µM JG-C3-98 (Cold 2) in the same neuron, with these thermal stimuli properly separated to reduce desensitization (Figure 2A). In the absence of the drug, the mean thermal threshold of cold thermoreceptors at two consecutive cold pulses remained unaltered (Figure 2B). Nevertheless, in the presence of JG-C3-98, we found that the mean thermal threshold of these neurons was shifted more than 2 °C to lower temperatures and the maximal cold-evoked response was slightly reduced (Figure 2C), suggesting that the activation of TASK-3-containing K2P channels by JG-C3-98 reduces the thermal sensitivity of cold thermoreceptors by decreasing the excitability of these sensory neurons.

Since TASK-1 channels are expressed in primary afferents, including nociceptive fibers, we asked whether JG-C3-98 affects the mechanically evoked responses of trigeminal neurons in culture. To this end, we also used a double-pulse protocol consisting of two consecutive applications of an extracellular hypoosmotic solution to induce membrane stretching. The second pulse was applied in the presence of control solution or a 100 µM JG-C3-98 solution (Figure 2D). To determine whether the positive responses to the hypoosmotic solution originated from mechanoreceptor neurons or from polymodal nociceptors, a pulse of 100 µM AITC, a potent TRPA1 agonist, was applied at the end of the experiment. This approach enables functional separation of neurons into AITC-insensitive and AITC-sensitive groups, with the latter potentially corresponding to polymodal nociceptive fibers. Under control conditions, we found that the mean maximal responses to both hypoosmotic stimulation pulses were indistinguishable in both AITC-sensitive and AITC-insensitive neurons (Figure 2E). In contrast, incubation with JG-C3-98 reduced the maximal response to hypoosmotic stimulation in both groups of sensory neurons (Figure 2F). These findings suggest that JG-C3-98 reduces the mechanosensitivity of primary sensory neurons, including AITC-sensitive polymodal nociceptive fibers.

### 3.3 JG-C3-98 shifts the thermal threshold of cold thermoreceptor neurons from dorsal root ganglia to lower temperatures

**FIGURE 3:**
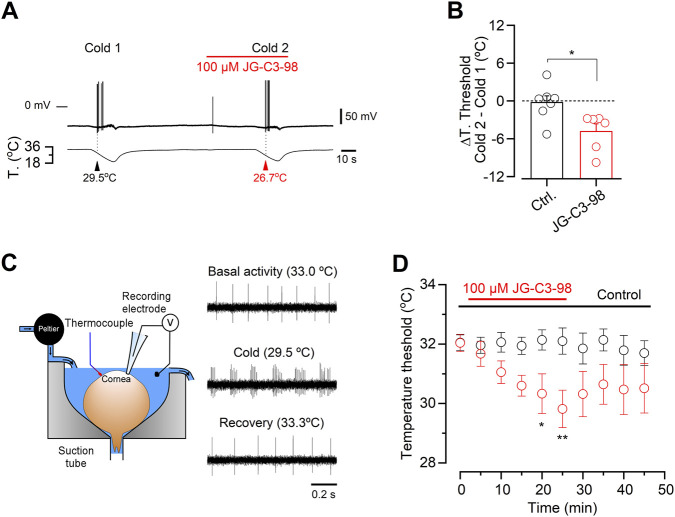
The TASK-1/TASK-3 activator JG-C3-98 shifts the thermal threshold of cold-evoked responses of primary sensory neurons. **(A)** Simultaneous recording of membrane potential (top trace) and bath temperature (bottom trace) during consecutive cooling ramps in a cold-sensitive DRG neuron recorded under current-clamp (I_hold_ = 0 pA). Two cold pulses were applied in control solution (control condition) or in the presence of 100 µM JG-C3-98 before and during the second pulse (JG-C3-98 condition); arrow heads indicate the thermal thresholds. **(B)** Bar and dots plot summarizing the effect of JG-C3-98 on the thermal threshold of cultured cold-sensitive neurons. Data points show the shift in the temperature threshold during the second cooling stimulus (T°. threshold Cold 2 - T°. threshold Cold 1) in control condition (n = 7) and in the presence of JG-C3-98 (n = 6); negative values indicate shifts toward lower temperatures (p = 0.0221). Statistical analysis was carried out using two-tailed Mann-Whitney test (*p < 0.05). **(C)** *Left panel,* Experimental arrangement of the *ex vivo* preparation used to record nerve terminal impulse (NTI) activity from corneal cold-sensitive nerve endings in isolated mouse eyes, as we described in (Parra et al., 2010). *Right panel,* Examples of 1 s of NTI activity recording from a corneal cold thermoreceptor neuron in basal conditions (ongoing activity), cold, and after rewarming (recovery). **(D)** Dot plot showing the mean temperature threshold of the cold-evoked responses in control corneal cold-sensitive neurons (open black dots, n ≥ 8 nerve endings) and the shift to lower temperatures observed in the nerve endings treated with 100 µM JG-C3-98 (open red dots; n ≥ 6 nerve endings). Statistical analysis was made using two-way ANOVA with Bonferroni post-hoc test (*p < 0.05; **p < 0.01).

Next, to further investigate the action of this TASK-1/3 activator on cold-sensitive neurons, we examined the effect of JG-C3-98 on cold-evoked firing in DRG cold thermoreceptor neurons, using the whole-cell patch-clamp technique. To this end, we used a double-pulse protocol wherein we studied the response to cold before and in the presence (or absence) of 100 µM of the drug in the same neuron under current-clamp (Figure 3A). In the absence of the drug, we found that the mean thermal threshold of cold-evoked firing at two consecutive cold pulses remained unaltered (Figure 3B). However, in the presence of JG-C3-98, the mean thermal threshold of cold-evoked firing induced by the second cold pulse was shifted to lower temperatures (Figure 3B), a result that is consistent with our findings using Ca^2+^ imaging in cultured trigeminal neurons (see Figures 2A–C). Interestingly, the resting membrane potential of these neurons was not significantly altered by JG-C3-98, and the number of action potentials during the cold-evoked response was slightly reduced (data not shown). These observations suggest that TASK channel activation by JG-C3-98 could be more effective during the development of cold-evoked depolarization than in resting conditions.

Additionally, because these findings could result from nonspecific alterations in the neuronal firing induced by off-target effects of JG-C3-98, we also investigated the impact of this compound on the passive and active membrane properties of cultured DRG neurons under current-clamp conditions. Although in a group of neurons JG-C3-98 reduces the input resistance and increases the rheobase current, consistent with the effect expected for a background potassium channel activator, we found that this compound does not impede the current-evoked firing in both cold-sensitive and cold-insensitive neurons, inducing only a slight reduction in the mean amplitude of the action potential (Supplementary Figure S4). As expected, the presence of a hump in the repolarizing phase was significantly more frequent in cold-insensitive neurons than in cold sensitive neurons (*p < 0.05, Fisher’s F exact test; data not shown).

To characterize the JG-C3-98-sensitive current in these neurons, we examined the effect of this compound on the whole-cell current evoked by voltage ramps from −120 to −30 mV in a group of cultured DRG neurons from control animals (Supplementary Figure S5). The JG-C3-98-sensitive current was obtained by subtracting whole-cell current traces recorded before and after incubation with the TASK channel opener, in the presence of 1 mM CsCl in the extracellular medium to block the I_h_ current in both conditions (Supplementary Figure S5A). The resulting current had a slightly outward rectification in cells where JG-C3-98 had an effect, showing a potentiation at −30 mV in four of 15 neurons recorded under these conditions (Supplementary Figure S5B). These findings are consistent with a previous report using an activator of TASK-3-containing channels in these sensory neurons (Liao et al., 2019), suggesting that the effect of activating TASK channels may not be homogenous among primary afferents.

Finally, to rule out the possibility that the thermal threshold of cold-sensitive neurons could be altered by an unspecific effect of JG-C3-98 on the main molecular entity responsible for cold-sensitivity in the somatosensory system, we also examined its effect on the TRPM8 channel. Using voltage ramps and a double-pulse cold stimulation protocol to activate TRPM8 expressed in HEK-293 cells, we found that JG-C3-98 does not block the cold-evoked current depending on this thermo-TRP channel (Supplementary Figure S6).

Altogether, these results suggest that activating TASK-1/TASK-3 channels using JG-C3-98 decreases the excitability of cold thermoreceptor neurons under physiological conditions and could be used to explore its effect on primary afferents *in situ* and *in vivo*.

### 3.4 JG-C3-98 shifts the activation threshold of corneal cold thermoreceptor neurons to lower temperatures

The cornea of the eye has the largest sensory nerve supply of all body tissues (Marfurt et al., 2010). Among these sensory fibers, trigeminal cold thermoreceptor neurons innervating the cornea that express TRPM8 and TASK-3 channels play a critical role in regulating ocular surface homeostasis. Thus, the nerve endings of these neurons not only act as cold sensors of the cornea but also function as accurate dryness detectors, maintaining ocular surface humidity by adjusting the basal tearing rate and the spontaneous blinking frequency (Parra et al., 2010; Quallo et al., 2015). Considering their relevance in ocular physiology, we explored the effect of the activation of TASK-3 channels *in situ*, using JG-C3-98 in an *ex vivo* preparation of excised eyes that preserve the integrity and functionality of the peripheral nerve endings innervating the corneal surface (Parra et al., 2010; Piña et al., 2019) (Figure 3C). To allow the drug to reach the nerve endings in the epithelial cell layer, we applied consecutive cold stimuli separated by 5 min and recorded NTI activity in cold-sensitive fibers, measuring cold-induced responses in both the control condition and in the presence of JG-C3-98 for 25 min. Using this approach, we found that the thermal threshold of the cold-evoked responses in these nerve endings was reversibly shifted to a lower temperature in the presence of the drug (Figure 3D), consistent with the effect we observed in cultured trigeminal and DRG cold thermoreceptor neurons using Ca^2+^ imaging and patch-clamp analysis.

These results suggest that the activation of TASK-3-containing channels with JG-C3-98 in intact corneal nerve endings increases the temperature decrease necessary to generate a significant cold-evoked response in these primary sensory fibers. This evidence supports the potential use of this strategy to reduce the excitability of cold thermoreceptors and modulate their function in different physiological and pathological processes where these neurons have a relevant role.

### 3.5 JG-C3-98-induced analgesia

**FIGURE 4:**
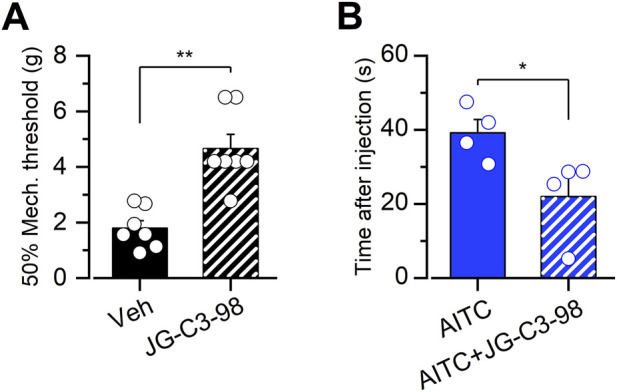
JG-C3-98 reduces the mechanical sensitivity and AITC-evoked responses *in vivo*. **(A)** Bar and dots graph summarizing the differences in the mechanical threshold in response to intraplantar administration of a single dose of the TASK-1/TASK-3 activator JG-C3-98 at 5 mM (p = 0.0026; n = 7 for each group). Statistical analysis was carried out using two-tailed Mann-Whitney test (**p < 0.01). **(B)** Bar and dots graph showing the differences in the duration of AITC-induced nocifensive behavior in response to intraplantar coadministration of a single dose of the TASK-1/TASK-3 activator JG-C3-98 at 5 mM (p = 0.0286; n = 4 for each group). Statistical analysis was carried out using two-tailed Mann-Whitney test (*p < 0.05).

Next, we evaluated the anti-nociceptive activity of JG-C3-98 using different pain models in mice. To this end, we first explored the effect of JG-C3-98 on the mechanical pain threshold, using Von Frey filaments. With this approach, we found that after 20 min of intradermal application of a single dose of JG-C3-98 in the hind paw, the force required to induce a mechanically evoked withdrawal response is doubled in treated animals (Figure 4A). Using a model of pain induced by intraplantar injection of the TRPA1 activator allyl isothiocyanate (AITC) (Moilanen et al., 2012), we found that the co-administration of JG-C3-98 also reduces the duration of the AITC-induced nociceptive response in mice (Figure 4B). Thus, these findings indicate that local activation of TASK-1/TASK-3 channels increases the threshold for mechanically evoked pain and effectively reduces AITC-evoked irritative pain responses in mice, supporting the potential applications of JG-C3-98 for pain alleviation.

### 3.6 JG-C3-98 reduces cold allodynia in a model of neuropathic pain

**FIGURE 5:**
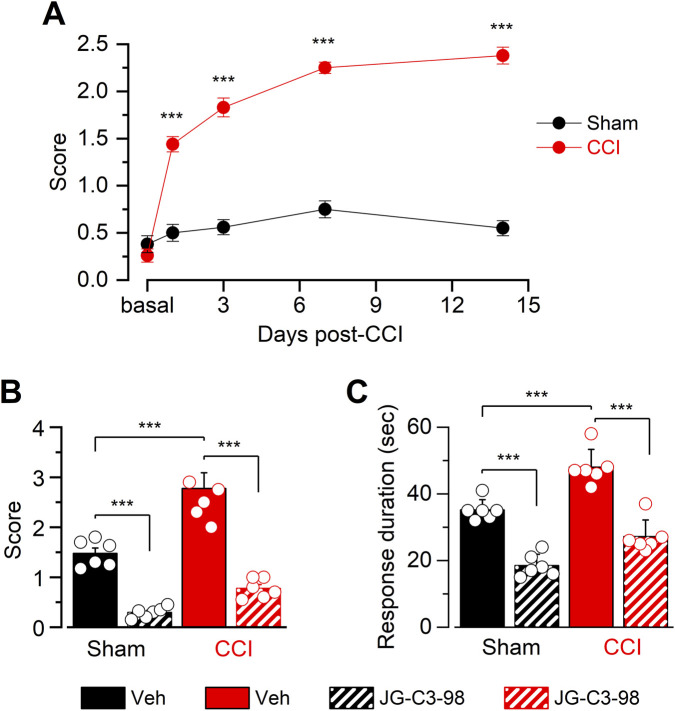
Pharmacological activation of TASK-1/TASK-3 by a single dose of JG-C3-98 reduces the nocifensive behavior induced by cold stimulation in sham and CCI mice. **(A)** Time course of cold-induced nocifensive behavior assessed by acetone response net score in mice at baseline (0 days), and at 1, 3, 7, and 14 days after injury affecting the L3, L4, and L5 DRG neurons’ function (see Methods). Red line and dots represent CCI animals, while black line and dots represent sham-operated mice; n = 6 animals for each group. **(B)** Bar and dots graph summarizing the nocifensive behavior after application of acetone to the plantar surface of the hind paw in sham and CCI animals, before and after pharmacological activation of TASK-1/TASK-3 by intraplantar injection of a single dose of 5 mM JG-C3-98. **(C)** Bar and dots graph showing the duration of nocifensive responses at B until the return to baseline; n = 6 animals in both conditions. Statistical analysis in **(A–C)** was made using two-way ANOVA followed by Bonferroni post hoc multiple-comparisons test (***p < 0.001).

Painful hypersensitivity to innocuous cold, or cold allodynia, is one of the most debilitating symptoms of neuropathic pain induced by peripheral nerve damage (Yin et al., 2015; Finnerup et al., 2021). Using chronic constriction injury (CCI) of the sciatic nerve as a model of peripheral nerve damage that induces cold allodynia, we investigated the effect of JG-C3-98 on this injury-triggered sensory alteration. Nocifensive responses to innocuous cold stimulation induced by acetone evaporation in the hind paw of CCI animals appear 1 day after nerve ligation and remain for at least 2 weeks after surgery (Figure 5A). On day seven, we tested the effect of the intraplantar injection of a single dose of JG-C3-98 on the nocifensive behavior evoked by acetone evaporation in the hind paw of injured and sham mice. We observed a marked reduction in the cold-evoked nocifensive responses in both CCI- and sham-operated animals following a single dose of this compound (Figures 5B,C), suggesting that JG-C3-98 may be also effective in reversing this frequently debilitating sensory alteration in neuropathic pain.

## 4 Discussion

The results presented here show that the novel activator of TASK-1 and TASK-3 channels, JG-C3-98, may serve as a pharmacological tool for modulating thermal and mechanical sensitivity of primary afferents in different somatosensory territories. Our findings suggest that JG-C3-98 has potential as an agent for regulating the threshold of mechanical pain, the duration of irritation-induced painful responses, and the cold-induced pain sensitivity in neuropathic pain, possibly restoring normal function of sensitized primary afferents under pathological conditions by acting on these two key brake K2P channels.

Inspired by common chemical features found in TASK channel modulators, including the presence of aromatic groups and nitrogen-containing heterocycles, the synthesis of JG-C3-98 led to the discovery of a novel and potent activator of TASK-1 and TASK-3 channels that could serve for the development of new antinociceptive compounds. As a molecular scaffold, JG-C3-98 could be further optimized, for example, in terms of solubility. Future derivatives may improve aqueous solubility by introducing polar or ionizable substituents without disrupting the aromatic scaffold required for channel activation. Among the known modulators of TASK-1 and TASK-3 channels, only a few activators contain cyclic scaffolds (see Supplementary Table S1), and these exhibit a very low Tanimoto similarity index with JG-C3-98, indicating limited structural resemblance. Therefore, this novel TASK-1/3 activator represents a new and distinct chemotype compared to those reported to date, with higher potency toward TASK-1 than TASK-3 (see Figures 1E,F). TASK-1 and TASK-3 share 54% amino acid identity, which increases to 80% in the pore region (Hall et al., 2025). To explore the differences in affinity for JG-C3-98 between the two channels, we focused on the HRE, where volatile anesthetics bind to activate TASK channels. The HRE sequences of TASK-1 and TASK-3 differ by a single residue, with a methionine present in TASK-1 instead of a leucine at position 247. We tested the L247M mutant in TASK-3, which resulted in reduced affinity for JG-C3-98, suggesting that this residue contributes to the activator binding site but is not responsible for the differences in affinity between TASK-1 and TASK-3. Nevertheless, we consider that the HRE, where JG-C3-98 interacts, is responsible for these differences in affinity. The HRE sequence motif forms the X-gate, a structural motif that constricts the M4 helices in TASK channels, thereby controlling the opening and closing of the channel pore. Although the crystal structures of both TASK-1 and TASK-3 exhibit the X-gate in a closed conformation (Hall et al., 2025; Rödström et al., 2020), it has been speculated that the X-gate of TASK-3 is subject to cholesterol modulation and that the closed X-gate observed in TASK-3 structures results from the high cholesterol concentrations used during sample preparation (Lin et al., 2024). Consequently, TASK-1 may predominantly adopt a closed X-gate conformation, and JG-C3-98 could therefore contribute more strongly to TASK-1 activation than to TASK-3 activation.

Through docking and molecular dynamics simulations, we investigated the interactions of JG-C3-98 in the TASK-1 (PDB ID: 6RV2) and TASK-3 (PDB ID: 8K1V) structures. The docking pose of JG-C3-98 differs between TASK-1 and TASK-3, with the 2-methylthiophene group pointing toward different subunits in each channel (see Figure 1B). The 2-methylthiophene group is located nearest to residue 247 in both channels, but it interacts with residue 247 in chain A of TASK-1 and in chain B of TASK-3. Moreover, the interactions differ between the two channels (see Supplementary Figure S2): while in TASK-1 channels JG-C3-98 does not interact with M247 (according to PLIP software) or interacts only weakly during the MD simulation (according to GetContacts software), in the TASK-3 channel, the interactions of JG-C3-98 with residue 247 are more frequent. This highlights that residue 247 in the TASK-3 channel is part of the JG-C3-98 binding site, which may account for the loss of activation observed in the L247M-TASK3 variant, whereas it may not be as relevant in TASK-1. Interestingly, the concentration response curve of TASK-3 channels with JG-C3-98 showed a potentiation of ∼20% of the maximal effect occurring in the submicromolar range, followed by the main activation component revealed at higher concentrations of this compound. Further studies are necessary to determine whether this finding results from multiple binding sites or state-dependent modulation of channel gating.

The expression and distribution patterns of TASK-1 and TASK-3 have been explored in primary somatosensory neurons, along with the co-localization patterns with key thermo-TRP channels in pain neurophysiology. In DRG immunostaining studies, TASK-1 is predominantly expressed in small-diameter neurons. In these sensory ganglia, 93% of the TASK-1-positive neurons express the nociceptive channel TRPV1, and approximately 43% of TRPV1-positive neurons co-express the TASK-1 channel (Pollema-Mays et al., 2013). Moreover, 58% of TASK-1-expressing neurons are IB4-positive, and 45% of the IB4 population co-expresses the TASK-1 channel. These results suggest that TASK-1 channels are expressed in TRPV1-positive neurons with an important overlap with IB4-expressing cells. In the study by Liao and coworkers, using *in situ* hybridization, the authors determined that TASK-3 channel mRNA is restricted to a subset of small-diameter primary sensory neurons (∼7%) in DRG. The expression of this channel is significantly larger in TG, reaching 14% of these sensory neurons (Liao et al., 2019). The co-expression of TRPM8 and TASK-3 has also been characterized. TRPM8-positive neurons constitute a relatively small subpopulation (∼8–15%) of primary somatosensory afferents, including low- and high-threshold cold-sensitive neurons present in DRG and TG neurons (Pertusa et al., 2023; González et al., 2017; Piña et al., 2024). In DRG neurons, in contrast to TASK-1, TASK-3 is strongly enriched at the transcript level in TRPM8-expressing neurons (∼140-fold in TRPM8-positive neurons than in TRPM8-negative neurons) (Morenilla-Palao et al., 2014), a distribution pattern that has also been reported in TG (Bhuiyan et al., 2024). In the same study, Morenilla-Palao and coworkers determined that ∼33% of TRPM8-positive neurons express the TASK-3 channel, and 68% of TASK-3-positive neurons co-express TRPM8. Interestingly, Liao and coworkers also found a large population (95%) of TASK-3-positive neurons expressing TRPM8, corroborating this link between TASK-3 channel expression in the TRPM8-positive population. In contrast, these authors also found low co-expression with the nociceptive channel TRPA1 in TASK-3-expressing neurons; furthermore, 50% of TASK-3-positive neurons co-express tyrosine hydroxylase (Liao et al., 2019), a marker of c-low-threshold mechanosensitive fibers (Brumovsky, 2016; Li et al., 2011). Remarkably, Liao and coworkers also found in DRG that 95% of TASK-3-expressing neurons co-express TASK-1, suggesting that TASK-1/TASK-3 heteromers can also be present in primary somatosensory neurons. Thus, although somewhat restricted and diverse, these expression and co-expression patterns are consistent with a TASK-1 channel being more represented in small-diameter neurons expressing nociceptive markers, and with TASK-3 expressed in small primary sensory neurons, including mainly TRPM8-positive cold-sensitive neurons and a particular subset of nociceptive fibers. These distribution patterns are consistent with the experimental findings presented here and with previous evidence supporting the role of TASK-1 and TASK-3 channels in pain neurophysiology, including the heterogeneous effects observed in some subgroups of primary sensory neurons.

Thus, in primary somatosensory neurons, the presence of TASK-1 and TASK-3 channels in nociceptors and thermoreceptors has been demonstrated, and their roles in pain physiology and pathophysiology are still emerging (Fan et al., 2022). Moreover, it has been reported that the expression of TASK-3 and TWIK-1 channels is downregulated in response to neuropathic lesions, with no significant changes in TASK-1 expression (Pollema-Mays et al., 2013; García et al., 2019). The present findings indicate that, despite potential reductions in protein levels that can occur in neuropathic conditions, novel TASK channel openers, such as JG-C3-98, may counteract hyperexcitability by activating the remaining functional channels following nerve injury. Additional studies are required to assess its potential analgesic effects across different somatosensory regions and forms of peripheral neuropathies in which these K2P channels may be involved and differentially altered, particularly considering the stronger activation effect of this compound on TASK-1 compared to TASK-3. Given their role as brake channels in primary afferents, this activator may also serve as a scaffold for the development of novel, more potent antinociceptive compounds that specifically target TASK channels.

Regarding TASK activators, CHET3 is a selective TASK-3 agonist that attenuates thermal hyperalgesia and mechanical allodynia in mice (Liao et al., 2019). In the elegant study by Liao and coworkers, systemic administration of CHET3 enabled the authors to evaluate the analgesic effects of TASK-3 channel activation. They found that this compound exhibits a predominantly peripheral anti-nociceptive effect in the tail-immersion and paw-pressure tests and attenuates mechanical allodynia and thermal hyperalgesia. These results are consistent with TASK-3’s relevant role in peripheral pain neurophysiology and align with our findings using local intradermal application of JG-C3-98 at the hind paw. Interestingly, the authors observed a rather small (∼18%) increase in current density at −30 mV in cultured primary sensory neurons upon CHET3 application, similar to our result with JG-C3-98. These findings suggest that even a relatively discrete potentiation of TASK-dependent currents in these neurons can effectively reduce their responses to physiologically relevant stimuli. Interestingly, CHET3 did not alter the resting membrane potential of DRG neurons, most likely because of the low density of TASK-dependent currents at resting conditions in these cells, consistent with our results using JG-C3-98. These results are compatible with the idea that activating TASK-channels could be more effective at more depolarized membrane potentials, even near the threshold for action potential firing, and/or during the development of the receptor potential induced by transduction channels’ activation. Additionally, TASK-1 and TASK-3 can form heteromeric channels in both heterologous expression systems and different native membranes including primary sensory neurons (Czirják and Enyedi, 2002; Berg et al., 2004; Kang et al., 2004; Kim et al., 2009; Turner and Buckler, 2013; Rinné et al., 2015; Liao et al., 2019). Given that this new activator acts on both channels, it may also activate heteromers, thereby expanding the compound’s relevant potential targets.

It is important to notice the potential use of JG-C3-98 to modulate cold-sensing fibers, most likely due to the high co-expression of TASK-3 with the cold-sensor channel TRPM8. The absence of TASK-3, or its activation above the basal level, alters the thermal thresholds of cold-sensitive neurons in a range of 4 °C. On one end, the thermal threshold for cold-evoked responses in cultured cold thermoreceptors from TASK-3 knockout mice is shifted toward warmer temperatures by approximately 2 °C (Morenilla-Palao et al., 2014). Conversely, activation of TASK-3 channels by JG-C3-98 results in a 2 °C shift to the opposite direction (see Figure 2). In a scenario in which injured animals develop cold allodynia, resulting from a 2 °C shift in the cold threshold of primary afferents toward warmer temperatures (González et al., 2017; Piña et al., 2024), JG-C3-98 may at least partially counteract this pathological condition. Another potential application of this compound could be on dry-eye sensation induced by peripheral nerve damage resulting from the hyperexcitability of cold-sensitive trigeminal afferents innervating the corneal surface (Bech et al., 2018; Piña et al., 2019). Further studies are necessary to determine whether activating TASK channels in these neurons with JG-C3-98 could be an effective tool to treat this pathological condition at its source, thereby increasing the activation threshold of the primary afferents that participate in pathological responses affecting ocular homeostasis and/or inducing corneal dysesthesias.

Nevertheless, it is important to note that our study does not include genetic loss-of-function approaches to directly demonstrate target engagement in native systems, thereby limiting the reach of our findings to some extent. Thus, additional studies will be necessary to determine whether the activation effects of JG-C3-98 are fully or partially abolished in animal models lacking functional expression of TASK-1, TASK-3, or both molecular targets of this novel compound. This approach would be informative not only in cultured primary sensory neurons but also in behavioral studies, considering thermal and mechanical sensitivity in different somatosensory territories. Besides, although JG-C3-98 does not activate other key K2P channels or TRPM8 at the concentration used in this work, other studies at lower concentrations of this compound will also be relevant to limit any interference from possible undesirable off-target effects within its window of action. Finally, we have centered our study on cold- and mechano-evoked responses of primary sensory neurons. Nevertheless, although we do not test the effect of JG-C3-98 on heat-evoked responses, the TASK-1/3 expression patterns suggest that this compound should also be useful to reduce nociceptive responses to heat stimulation. Further studies are necessary to expand the repertoire of somatosensory input that could be modulated by this compound, including noxious thermal pain, not only under physiological but also under pathological conditions.

In addition to TASK channels, other K2P channels have been pointed out as relevant players in the excitability of primary sensory neurons. Among them, TREK channels have been recognized as relevant molecular targets in pain neurophysiology (Bayliss and Barrett, 2008; Ávalos Prado et al., 2022). TREK-1 knockout mice show increased heat- and mechanical-pain sensitivity (Alloui et al., 2006). The double-knockout mice for the TREK-1/TREK-2 and TRAAK channels exhibit enhanced painful thermal sensitivity following axonal injury (Noël et al., 2009), while TREK-2 plays an important role in controlling firing activity in nociceptive fibers (Pereira et al., 2014). In nociceptors, TREK-2 and TRESK form functional heterodimeric channels (Lengyel et al., 2020), and the TREK channel opener BL-1249 reduces formalin-induced mechanical allodynia and hyperalgesia (García et al., 2021). Moreover, light-induced analgesia mediated by TRAAK channel activation in nociceptors has been recently described (Bied et al., 2026). Thus, given their critical role in physiological and pathological pain, K2P channels have been highlighted as key targets in the development of more effective pain medications, and activators of TASK channels, such as JG-C3-98, or novel multi-target openers of several K2P channels, emerge as promising tools. Considering the impact of TASK channels on the fine-tuning of the electrical response in primary afferents, a specific activator of TASK-1/3 channels that reduces its net excitability by counteracting the depolarizing effect of transduction channels at the peripheral nerve endings is very appealing.

In conclusion, these experimental findings support pursuing and optimizing modulators targeting these pivotal channels as an effective strategy to reversibly reduce excitability in primary afferents. This approach could be relevant not only under physiological conditions but also in peripheral neuropathies, where these channels play a significant role.

## Data Availability

The original contributions presented in the study are included in the article/Supplementary Material, further inquiries can be directed to the corresponding authors.
