# Supplementary material for: The TASK-1 and TASK-3 activator JG-C3-98 attenuates cold and mechanical responses in primary somatosensory neurons
Source: Front Pharmacol. 2026 Jun 9;17:1844406. doi: 10.3389/fphar.2026.1844406 (PMC13286800; doi:10.3389/fphar.2026.1844406)
Supplement: Supplementary file 1 [file DataSheet1.pdf]

Supplementary Material

1. Supplementary Figures and Legends

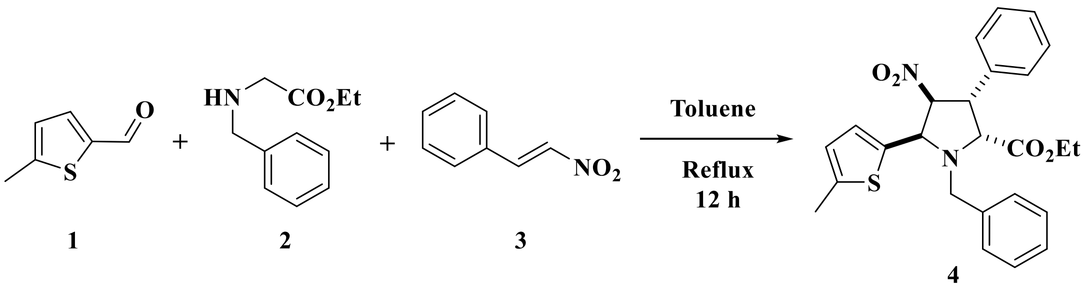

1 2 3 4

Supplementary Figure 1. Schematic representation of the reaction protocol to obtain JG-C3-98. Starting materials were mixed and under reflux conditions in a 1:1:1.5 eq. ratio of **1**, **2**, and **3**, respectively. The crude product was purified by column chromatography, obtaining a racemic mixture of stereoisomers of **4.**

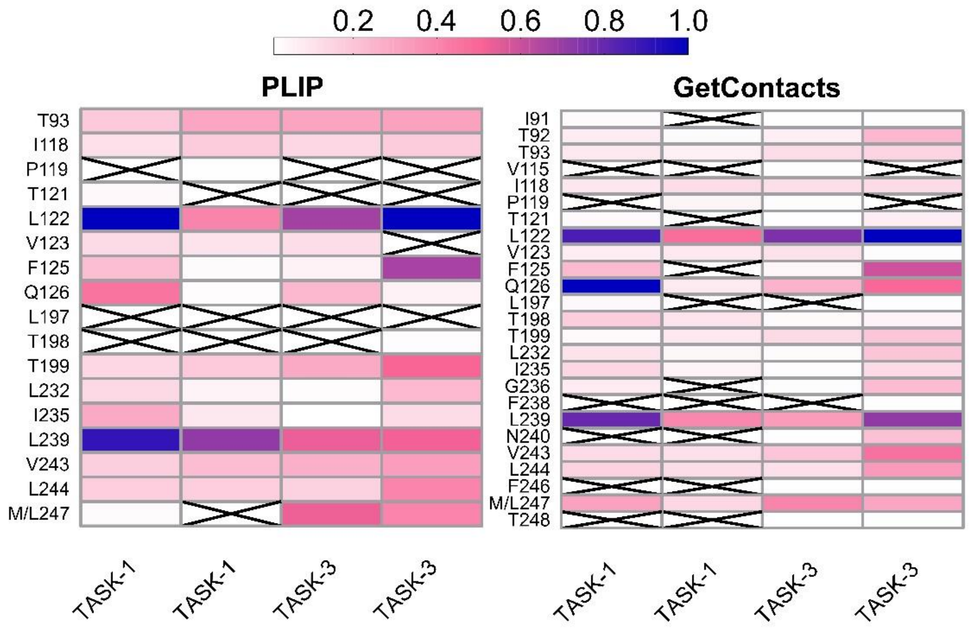

Supplementary Figure 2. Non-covalent interactions of JG-C3-98 with TASK-1 and TASK-3. Comparison of non-covalent interactions between the ligand JG-C3-98 and the TASK-1 and TASK-3 channels obtained using the software tools PLIP (left panel) and GetContacts (right panel) (see Methods). Rows represent the residues from subunits A and B of each channel, while columns correspond to each channel-subunit combination. The color scale indicates the relative frequency of interaction between each residue and the ligand, normalized from 0 (no interaction) to 1 (interaction present in all replicas). Cells marked with an "X" indicate the absence of detected interactions; cells from white to dark blue indicate at least one interaction.

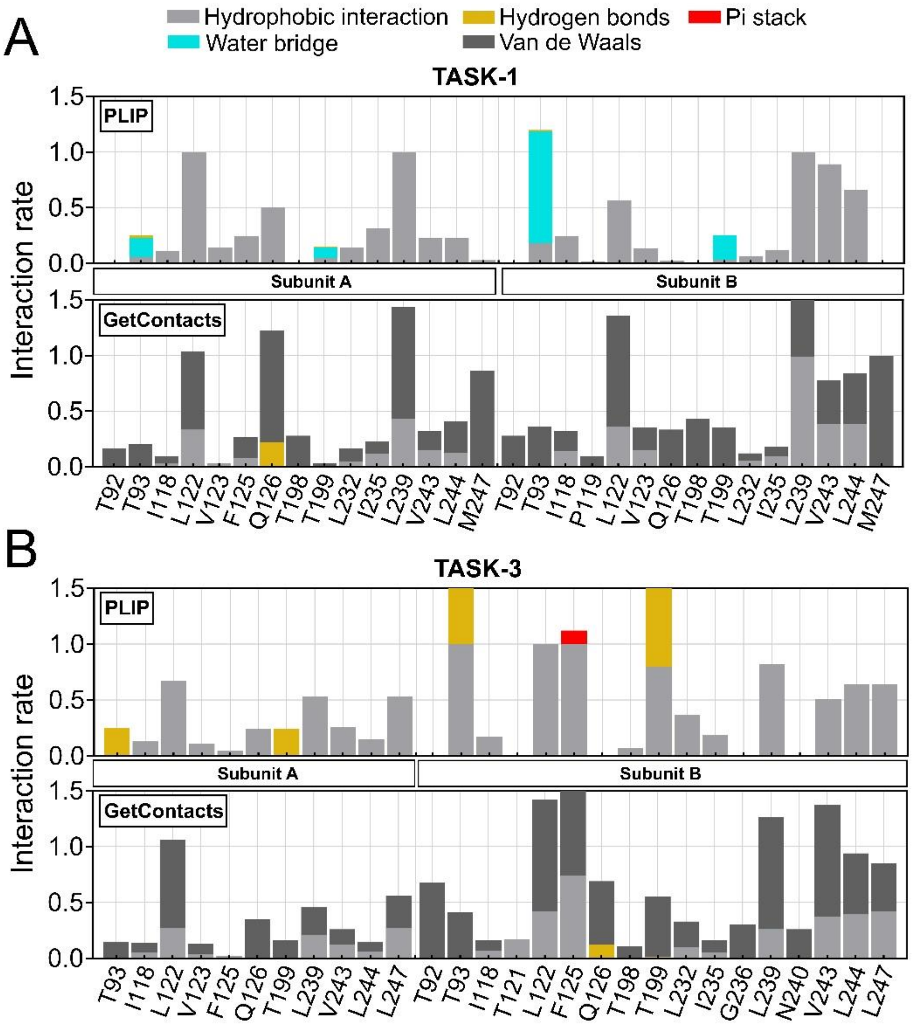

Supplementary Figure 3. **Nature of the interactions between JG-C3-98 and the residues located within ≤ 5 Å. A-B.** The interactions were calculated using PLIP and Get Contacts, for TASK-1 (A) and TASK-3 (B). In GetContacts, hydrophobic contacts correspond to a chemically defined subset of close-range atomic contacts that geometrically overlap with van der Waals interactions.

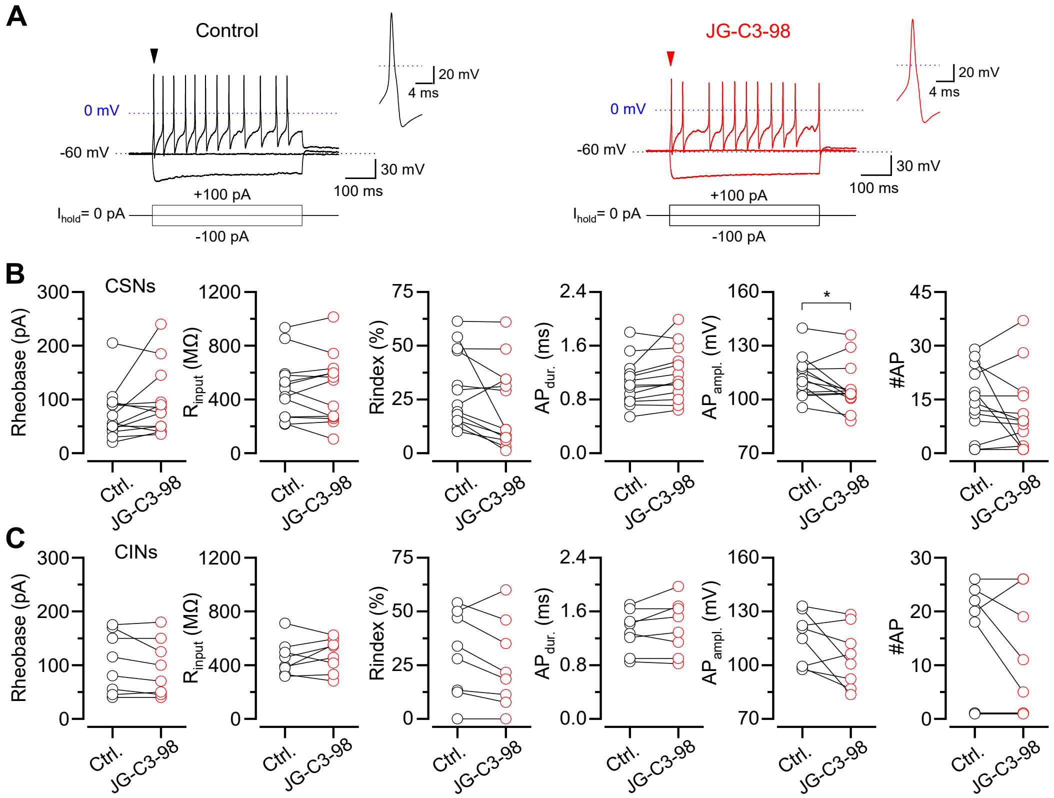

Supplementary Figure 4. Electrophysiological properties of primary sensory neurons from DRG in control conditions and in the presence of JG-C3-98. **A.** Voltage responses to 500 ms hyperpolarizing and depolarizing current pulses (I_{ext} at bottom) from a representative cold-sensitive DRG neuron in control conditions and in the presence of 100 µM JG-C3-98. *Insets*, First action potential of the I_{ext}-evoked firing in the neuron in control (black arrowhead) and JG-C3-98 (red arrowhead). **B-C**. Dot plots showing rheobase current, input resistance, rectification index, action potential duration, action potential amplitude, and number of action potential evoked at 1.5x rheobase current of individual cold-sensitive (B) and cold-insensitive (C) DRG neurons in control condition and in the presence of JG-C3-98 (n= 13 cold-sensitive neurons and n= 8 cold-insensitive neurons. Statistical significance was assessed with two tailed paired Student *t*-test; *p= 0.0395).

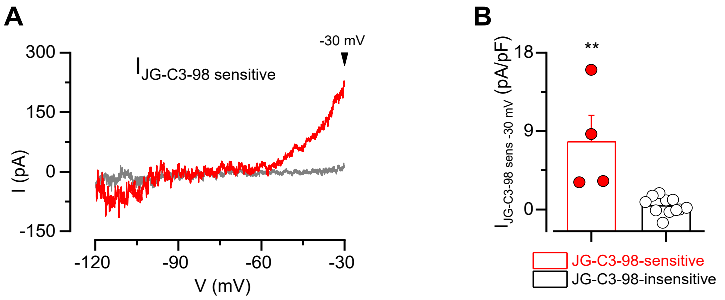

Supplementary Figure 5. JG-C3-98 potentiates the membrane current in a group of cultured DRG neurons. A. Representative traces showing the JG-C3-98-sensitive current in response to depolarizing ramps from -120 to -30 mV (200 mV/s), obtained from the digital subtraction of the mean whole-cell currents in control solution and in the presence of 100 μM JG-C3-98. The red trace corresponds to a JG-C3-98-sensitive neuron, and the gray trace to a JG-C3-98-insensitive neuron. **B.** Dot and bar plot summarizing the density of the JG-C3-98-sensitive current at -30 mV (black arrowhead in A) in sensitive (n= 4, one cold-sensitive and three cold-insensitive; red dots and bar) and insensitive (n= 11, six cold-sensitive and five cold-insensitive; open black dots and bar) DRG neurons; p= 0.0050. Statistical significance was assessed with two-tailed Mann Whitney test (**p<0.01).

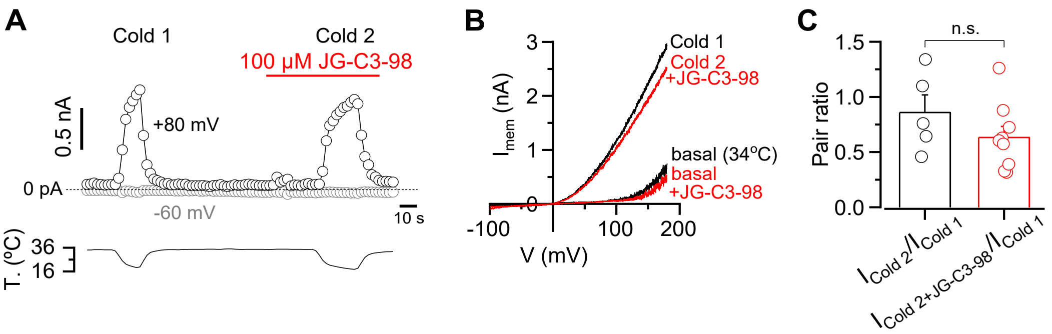

Supplementary Figure 6. JG-C3-98 does not block the TRPM8 channel. A. Quantification of representative recordings of whole-cell cold-evoked currents measured at +80 and -60 mV (open black and open gray circles, respectively) in HEK-293 transfected with mTRPM8 channels. **B.** I-V relationship of TRPM8 currents in control (basal and Cold 1, black traces) and JG-C3-98 (basal + JG-C3-98 and Cold 2 + JG-C3-98, red traces) conditions of the cell in A. **C.** Bar graph and data points of the pair ratio of cold-evoked current at +80 mV in control ($I_{Cold\ 2}/I_{Cold\ 1}$, open black dots and bar) and with JG-C3-98 in the second cold pulse ($I_{Cold\ 2+JG\text{-}C3\text{-}98}/I_{Cold\ 1}$, open red dots and bar); n= 5 cells for control and n= 9 for JG-C3-98 (p= 0.1898). Statistical significance was assessed using two-tailed Mann-Whitney test (n.s. p>0.05).

2. Supplementary Table and Legend

Channel	Molecule	**Activity increase**	Cell line	Article DOI	Image	Tanimoto index %
TASK-1	JG-C3-98	EC_{50} = 13±5 µM<br>100 µM → ≈500%↑	HEK-293	This article	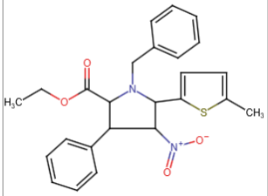	-
TASK-3	JG-C3-98	EC_{50} = 34±2 µM<br>100 µM → ≈100%↑	HEK-293	This article	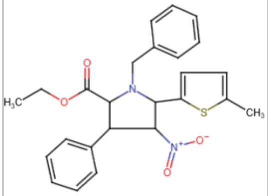	-
TASK-1	Halothane	300 µM → ≈50%↑	HEK-293	10.1074/jbc.M200502200	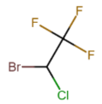	3.12%
TASK-3	Halothane	300 µM → ≈130%↑	HEK-293	10.1074/jbc.M200502200	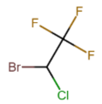	3.12%
TASK-1	2,2,2-tribromoethanol (TBE)	4400 µM → ≈3531%↑	FRT epithelial cells	10.1124/mol.117.108290	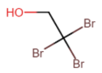	8.47%
TASK-3	2,2,2-tribromoethanol (TBE)	EC_{50} = 295 µM	FRT epithelial cells	10.1124/mol.117.108290	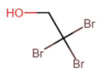	8.47%

TASK-1	ONO-RS-082	10 µM → ≈50%↑	COS-7	10.1056/NEJMoa1211097	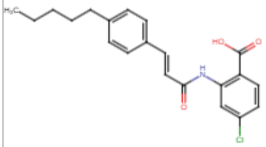	12.09%
TASK-1	Riociguat	10 µM → ≈1.8-fold↑	tsA201	10.1113/JP277275	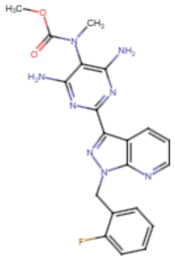	14.14%
TASK-3	1,1,1,3,3,3-hexafluoropropanol (HFP)	EC_{50} = 1942 µM	FRT epithelial cells	10.1124/mol.117.108290	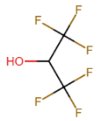	3.17%
TASK-3	2,2,2-trichloroethanol (TCE)	EC_{50} = 1077 µM	FRT epithelial cells	10.1124/mol.117.108290	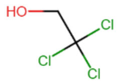	6.67%
TASK-3	CBr4	EC_{50} = 16 µM	FRT epithelial cells	10.1124/mol.117.108290	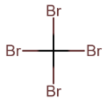	3.51%
TASK-3	CCl4	EC_{50} = 289 µM	FRT epithelial cells	10.1124/mol.117.108290	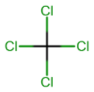	3.51%
TASK-3	CHET3	EC_{50} = 1.4±0.2 µM	HEK-293	10.1126/scitranslmed.aaw8434	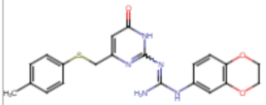	7.62%

TASK-3	Chloral hydrate	EC_{50} = 7141 μM	FRT epithelial cells	10.1124/mol.117.108290	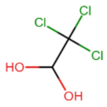	4.92%
TASK-3	Cinalukast	EC_{50} < 10 μM	U-2 OS	10.1016/j.bbrc.2019.09.093	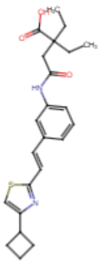	13%
TASK-3	Desflurane	EC_{50} = 433 μM	FRT epithelial cells	10.1124/mol.117.108290	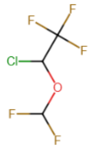	4.41%
TASK-3	Flufenamic Acid (FFA)	100 μM → 24%↑	tsA201	10.1124/mol.113.090530	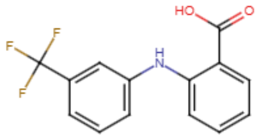	12.66%
TASK-3	Isoflurane	EC_{50} = 363 μM	FRT epithelial cells	10.1124/mol.117.108290	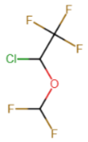	4.41%
TASK-3	NPBA	10 μM → ≈6-fold↑	CHO-K1	10.1124/mol.118.115303	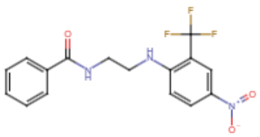	19.28%

TASK-3	Pranlukast	$EC_{50} < 10$ µM	U-2 OS	10.1016/j.bbrc.2019.09.093	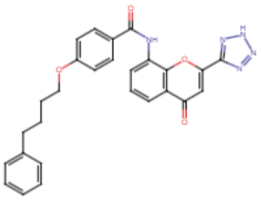	12.50%
TASK-3	Sevoflurane	$EC_{50} = 265$ µM	FRT epithelial cells	10.1124/mol.117.108290	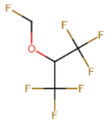	4.48%
TASK-3	Terbinafine	1 µM → 30%↑	tsA201 cells	10.1016/j.bbrc.2017.09.002	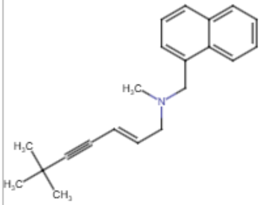	11.90%
TASK-3	Zafirlukast	$EC_{50} = 2.5$ µM	U-2 OS	10.1016/j.bbrc.2019.09.093	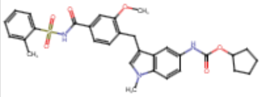	13.51%

Supplementary Table 1. Supplementary table summarizing the information about different available activators TASK-1 and TASK-3 channels, including the name of the molecule, percentage of activation and/or EC_{50} values, cell line where it was studied, reference (article DOI), molecular structure, and Tanimoto index %.
